# From the Bottom-Up: Chemotherapy and Gut-Brain Axis Dysregulation

**DOI:** 10.3389/fnbeh.2018.00104

**Published:** 2018-05-22

**Authors:** Juliana E. Bajic, Ian N. Johnston, Gordon S. Howarth, Mark R. Hutchinson

**Affiliations:** ^1^Discipline of Physiology, School of Medicine, Faculty of Health Sciences, University of Adelaide, Adelaide, SA, Australia; ^2^School of Psychology, The University of Sydney, Sydney, NSW, Australia; ^3^School of Animal and Veterinary Sciences, University of Adelaide, Adelaide, SA, Australia; ^4^Department of Gastroenterology, Women's and Children's Hospital, North Adelaide, SA, Australia; ^5^Centre of Excellence for Nanoscale Biophotonics, The University of Adelaide, Adelaide, SA, Australia

**Keywords:** chemotherapy-induced cognitive impairment, mucositis, chemotherapy-induced gut toxicity, gut-brain axis, microbiota

## Abstract

The central nervous system and gastrointestinal tract form the primary targets of chemotherapy-induced toxicities. Symptoms associated with damage to these regions have been clinically termed chemotherapy-induced cognitive impairment and mucositis. Whilst extensive literature outlines the complex etiology of each pathology, to date neither chemotherapy-induced side-effect has considered the potential impact of one on the pathogenesis of the other disorder. This is surprising considering the close bidirectional relationship shared between each organ; the gut-brain axis. There are complex multiple pathways linking the gut to the brain and vice versa in both normal physiological function and disease. For instance, psychological and social factors influence motility and digestive function, symptom perception, and behaviors associated with illness and pathological outcomes. On the other hand, visceral pain affects central nociception pathways, mood and behavior. Recent interest highlights the influence of functional gut disorders, such as inflammatory bowel diseases and irritable bowel syndrome in the development of central comorbidities. Gut-brain axis dysfunction and microbiota dysbiosis have served as key portals in understanding the potential mechanisms associated with these functional gut disorders and their effects on cognition. In this review we will present the role gut-brain axis dysregulation plays in the chemotherapy setting, highlighting peripheral-to-central immune signaling mechanisms and their contribution to neuroimmunological changes associated with chemotherapy exposure. Here, we hypothesize that dysregulation of the gut-brain axis plays a major role in the intestinal, psychological and neurological complications following chemotherapy. We pay particular attention to evidence surrounding microbiota dysbiosis, the role of intestinal permeability, damage to nerves of the enteric and peripheral nervous systems and vagal and humoral mediated changes.

## Introduction

The chemotherapy experience is associated with powerful psychological, neurological and somatic side-effects. Cancer diagnosis and the complications arising from treatment induce anxiety and depression, fatigue, pain, and cognitive impairments while patients struggle to maintain hope for recovery and continue normal daily functions, routines and roles (Kuzeyli Yildirim et al., 2005; Downie et al., 2006; Chan et al., 2014). Due to the non-selective and systemic nature of most chemotherapy drugs, they also target healthy, rapidly-dividing non-malignant cells. The regions of the body most susceptible to the unwanted toxicities of chemotherapy exposure are the gastrointestinal tract (GIT) and the central nervous system (CNS)—the gut and brain. Many chemotherapy drugs are small enough to readily cross the blood-brain barrier and result in molecular, structural and functional changes within the CNS, manifesting as cognitive changes in a subset of patients (Wigmore, 2012). Outside of the CNS, the cells of the GIT are particularly vulnerable to damage following chemotherapy exposure. In particular, epithelial cells within the mucosal layer lining the alimentary tract form prime targets due to chemotherapy drugs targeting proliferating enterocytes (Sonis, 2004). Although the gut and the brain appear disparate, they are intimately connected. The complex network of pathways linking the gut to the brain will be discussed in more detail below as we present mechanisms by which chemotherapy results in gut-brain axis dysregulation.

**Figure 1:**
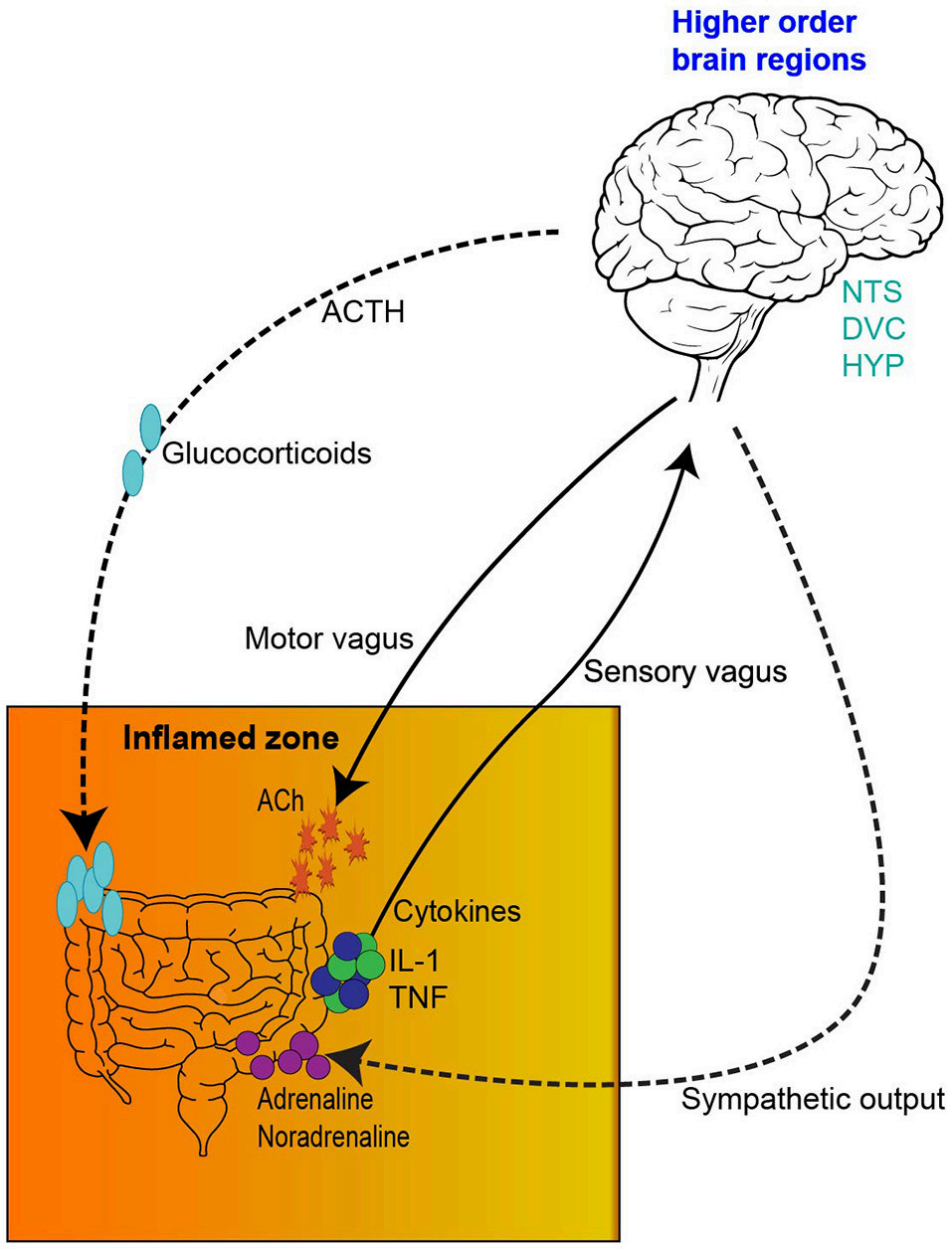
The inflammatory reflex. (1) The inflamed zone represents tissue damage, infection and ischemia. (2) Increased expression of inflammatory mediators and cytokines, such as Il-1β and TNF-α are released by cells in the inflamed zone. (3) Cytokines activate primary afferent neurons within the vagal sensory ganglia. (4) Afferent visceral signals are relayed to nuclei in the dorsal vagal complex (DVC), such as the nucleus tractus solitarius. (5) Visceral information is further relayed from the DVC to higher order brain regions, such as the hypothalamus, hippocampus and forebrain. (6) Activation of efferent vagal motor activity inhibits cytokine synthesis. (7) Hypothalamus activation stimulates the release of adrenocorticotrophic hormone from the pituitary gland, initiating a humoral anti-inflammatory pathway. (8) Sympathetic outflow can increase localized adrenaline and noradrenaline expression further suppressing inflammation. IL-1β, interleukin-1 beta; TNF-α, tumor necrosis factor—alpha; DVC, dorsal vagal complex; NTS, nucleus tractus solitarius; HYP, hypothalamus; HIP, hippocampus; FB, forebrain; ACTH, adrenocorticotrophic hormone.

This network has a bidirectional relationship. For instance, psychological and social factors have the ability to influence motility and digestive function, symptom perception, behaviors associated with illness and the pathological outcome (Bhatia and Tandon, 2005). On the other hand, visceral pain affects central pain perception and pathways, mood and behavior (Chakiath et al., 2015). Importantly, systemic and gut immunity is tightly regulated by the inflammatory reflex and cholinergic anti-inflammatory pathway (Tracey, 2002; Pavlov and Tracey, 2012). Integral components of the inflammatory reflex include innate immune cell activation, release of inflammatory mediators, such as cytokines, vagal innervation and responses from higher order brain regions, such as the nucleus tractus solitarius. Vagal innervation is of particular importance in the chemotherapy setting as it is pivotal in the transmission of chemo and mechanosensory information from the gut to the brain (Figure 1; Goehler et al., 2000; Tracey, 2002). In this sense, proinflammatory mediators and cytokines, such as interleukin-1 (IL-1) and tumor necrosis factor (TNF) activate primary afferent nerve fibers within the vagal sensory ganglia. Vagal ganglia signal several nuclei of the dorsal vagal complex responsible for the integration of visceral sensory input and relay information to higher order brain regions like the hypothalamus, hippocampus and forebrain. Coordinated autonomic and behavioral responses are initiated to assist in restoration of homeostasis. Importantly, efferent vagal motor activity inhibits cytokine synthesis, creating the inflammatory reflex effect. Humoral anti-inflammatory pathways can be activated, stimulating the release of adrenocorticotrophin hormone. Sympathetic outflow can also increase localized adrenaline and noradrenaline expression and further suppress inflammation. The activation of these innate components of the inflammatory reflex, including the vagally-mediated cholinergic efferent output, ultimately results in the regulation of systemic and localized inflammation, having important implications in gut immunity (Figure 1). A more comprehensive outline of the inflammatory reflex has been reviewed elsewhere (Tracey, 2002; Pavlov and Tracey, 2012).

**Figure 2:**
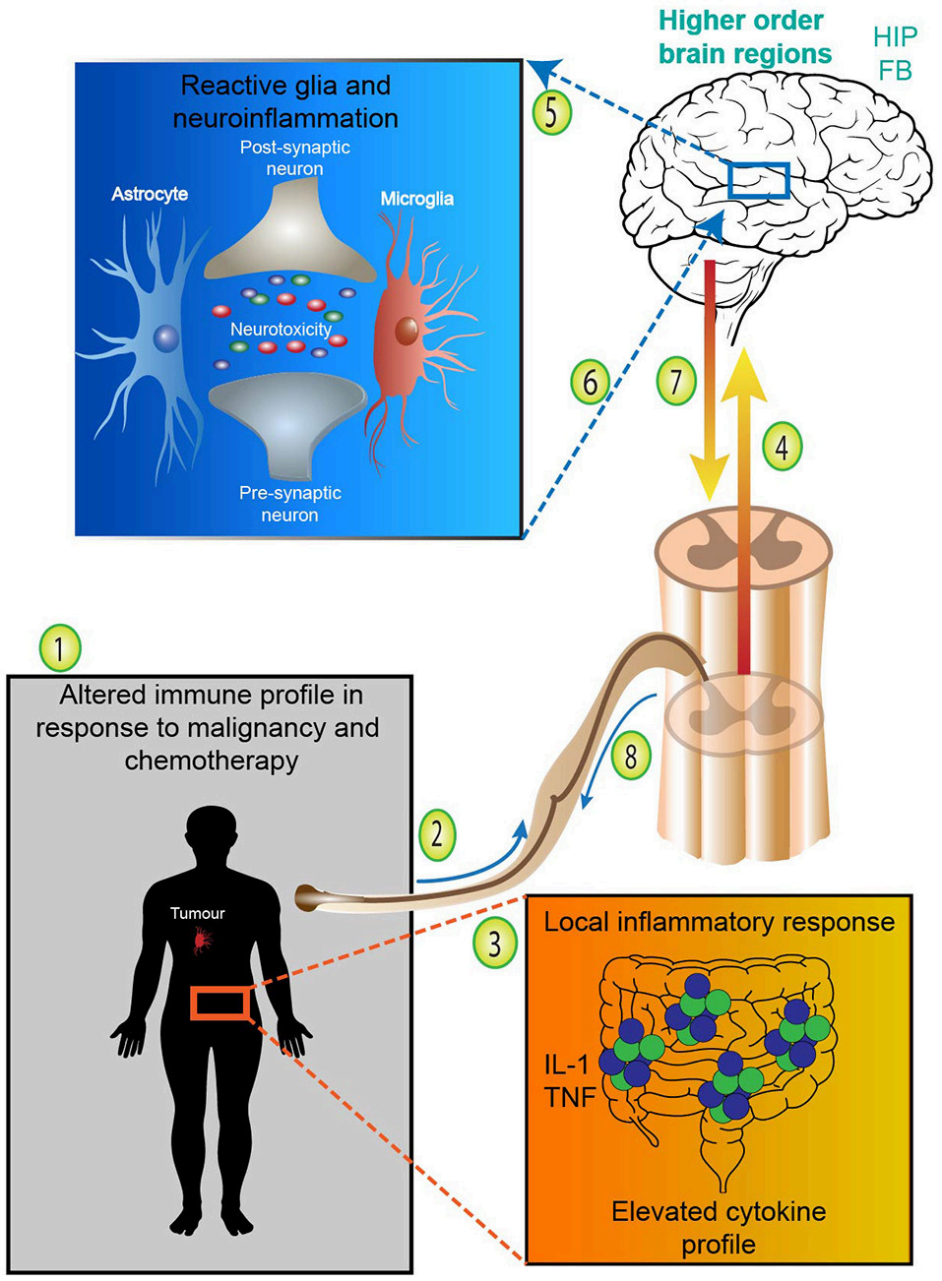
Adapted from Dodds et al. (2016). Neuroimmunological complications arising from cancer and chemotherapy treatment. (1) Cancer patients undergoing chemotherapy treatment express an altered immune profile with increases in proinflammatory cytokines. (2) Systemic proinflammatory cytokines and mediators, such as IL-1 and TNF released either from the malignancy or treatment-associated toxicities access the brain directly via leaky circumventricular organs or (3) indirectly via neural transmission. (4) Systemic or localized proinflammatory mediators and cytokines signal higher order brain regions and (5) activate microglia and astrocytes. Reactive glia undergo morphological changes and overproduce proinflammatory mediators whilst reducing anti-inflammatory output—resulting in neurotoxicity and neuroinflammation. (6) In particular brain regions primed glia and neuroinflammation influence behaviors involving cognition and contribute to various neurodegenerative diseases. (7) Centrally derived neurogenic inflammation and descending signaling in the spinal cord (8) contributes to the exacerbation of peripheral inflammatory conditions and exaggerated pain states. Therefore, peripheral-to-central innate immune signaling represents a plausible mechanism meditating chemotherapy-induced gut toxicity and neurological changes. FB, forebrain; HIP, hippocampus; IL-1, interleukin-1; TNF, tumor necrosis factor.

Additionally, activation of the neuroimmune system via glial priming and neurogenic inflammation further complicates immune to brain signaling. Although glial cells are non-neuronal cell types which can be found in the CNS and periphery, such as oligodendrocytes and Schwann cells, for the remainder of this manuscript we specifically refer to microglia and astrocytes. For an in depth analysis of glial priming and neuroinflammation several excellent reviews exist (Araque et al., 1999; Bains and Oliet, 2007; He and Sun, 2007; Allen and Barres, 2009; Capuron and Miller, 2011; Parpura et al., 2012; Dodds et al., 2016). Nonetheless, to illustrate this point in the context of cancer and chemotherapy, inflammation (either centrally or locally derived from either the malignancy or chemotherapy) and the release of proinflammatory cytokines signals the brain and activates neuroimmunological cells, glia (Figure 2). Proinflammatory cytokines access the brain either directly via leaky circumventricular organs or indirectly via a neural route (e.g., vagal transmission). Microglia and astrocytes form an integral part of the tri- and tetra-partite synapses and form a close bidirectional relationship with neurons; the neuroimmune interface which has wide implications in central health and disease (Allen and Barres, 2009; Graeber and Streit, 2010; Grace et al., 2014; Dodds et al., 2016). Reactive glia undergo morphological changes and overproduce proinflammatory mediators whilst reducing anti-inflammatory output (O'Callaghan et al., 2008; Agrawal and Yong, 2011). Ultimately, glial reactivity results in a neuroinflammatory environment whereby neurotoxicity causes damage to surrounding tissues and neurons (Eikelenboom et al., 2006; Bilbo et al., 2012; Laskaris et al., 2015). Centrally derived neurogenic inflammation and signaling also contributes to the exacerbation of peripheral inflammatory conditions. Although glial reactivity may begin with beneficial intentions by responding to insults (disease, trauma, infection or drug exposure), glia may remain in a primed state and be sensitized even after the initial insult has resolved, eliciting an exaggerated immune responses (Figure 2). Critically, in particular brain regions primed glia and neuroinflammation influence behaviors involving cognition and are involved in the pathogenesis of various neurodegenerative diseases and pathological pain states (McGeer et al., 1988; Eikelenboom et al., 2006). Due to the altered immune profile of cancer and chemotherapy patients, it has been suggested that neuroinflammatory processes may be contributing to the cognitive deficits often experienced by this patient group (Myers, 2009; Johnston, 2014). This form of innate immune (peripheral-to-central) signaling represents a plausible mechanism meditating chemotherapy-induced gut toxicity and neurological changes (Figure 2).

Following on from this, it is not surprising that interactions between the immune system and the brain become dysregulated under cancer and chemotherapy conditions. Further, recent evidence has highlighted the impact gut commensal bacteria has in both central and peripheral development and health (Feng et al., 2018). Importantly, dysbiosis (microbial imbalance/maladaptation) and gut-brain axis dysfunction have been associated with functional gut disorders having negative effects on cognition (Jones et al., 2006; Frank et al., 2007). Previously, research has focussed on a single pathological manifestation of chemotherapy exposure, for example gut toxicity or regional structural brain changes (Keefe et al., 1997; Christie et al., 2012). Such studies have failed to consider the *indirect* effects of simultaneously occurring treatment-induced toxicities, which may be contributing to the primary pathology under investigation. Consequently, we hypothesize that chemotherapy treatment causes severe and prolonged psychosocial impacts on the survivor. Furthermore, we suggest that the gut-brain axis is an important mediator of a diverse range of cognitive and emotional disorders similar to those experienced by cancer survivors. Here, we will determine whether chemotherapy affects the gut-brain axis and present several key stages. Following on from this, we suggest that the psycho-social side effects of chemotherapy treatment could be caused by the effects of chemotherapy on the gut-brain axis.

Following a brief analysis of gut-brain communication, we will review some key studies linking gut-brain axis dysregulation to specific psychiatric disorders, highlighting similarities between these conditions and the chemotherapy setting. From the bottom-up (GIT to the brain) we will examine chemotherapy-induced gut and central changes and present several mechanisms meditating gut-brain axis dysregulation in the chemotherapy setting; focussing on the microbiome, intestinal integrity, peripheral neuron and enteric nervous system (ENS) dysfunction. Finally we will address the role vagal-, neural-, and humoral-mediated responses may play in these complex chemotherapy-induced pathological conditions. Overall, we aim to illustrate the complex role gut-brain axis dysregulation plays in shaping neurological changes associated with chemotherapy exposure.

## Gut brain crosstalk

**Figure 3:**
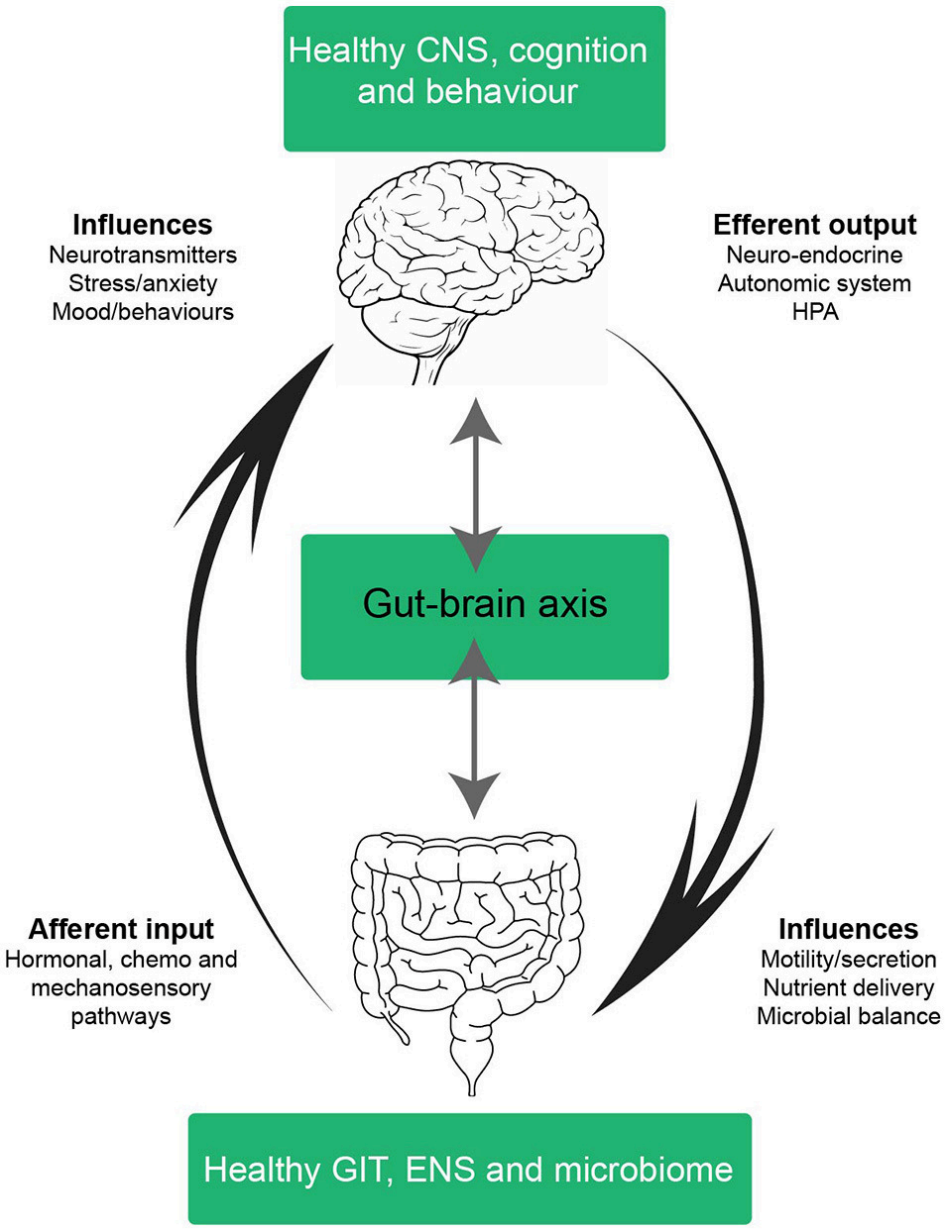
Schematic of a healthy gut-brain axis. The arrows highlight the bidirectional nature of the gut-brain axis; in a balanced system mechanisms from the bottom-up (and vice versa) exist in cohesion. In a healthy system, the gut-brain axis integrates information from many systems; the central nervous system (CNS), autonomic nervous system (ANS), enteric nervous system (ENS), neuroendocrine, enteroendochrine, neuroimmune, and hypothalamic-pituitary axis (HPA). The complex bidirectional communication pathways and systems shared between the gut and the brain maintain health and homeostasis in the CNS, GIT, and microbiota. Efferent signals from the brain involving neuro-endocrine, autonomic and HPA outputs influence motility, secretion, nutrient delivery and microbial balance in the GIT. Whilst afferent inputs from the GIT, such as intestinal hormones, cytokines and sensory perceptions influence neurotransmitter expression, stress, anxiety, mood and behavior. CNS, central nervous system; ANS, autonomic nervous system; ENS, enteric nervous system; HPA, hypothalamic-pituitary axis; GIT, gastrointestinal tract.

Since Pavlov's Nobel Prize-winning discovery on the role neural innervation plays in gastric secretion—the first functional evidence connecting the gut and brain—our understanding of the pathways connecting the CNS and the GIT have significantly advanced (Keller and William, 1950). The multiple bidirectional pathways responsible for controlling signaling from the brain to the gut and vice versa have been extensively reviewed and is outside the scope of this manuscript (Mayer, 2011; Al Omran and Aziz, 2014; Carabotti et al., 2015; Furness, 2016). The complexity of this network is best appreciated in its ability to integrate information from a variety of systems encompassing the central, autonomic and enteric nervous systems (including the influence of the intestinal microbiota), whilst simultaneously considering neuroendocrine, enteroendocrine and neuroimmune input (summarized in Figure 3; Carabotti et al., 2015). A brief analysis of bottom-up and ENS mechanisms is necessary to appreciate the systems by which the integration of these pathways influence behavior and impact central comorbidities in disorders of the gut. We begin this section from the bottom-up; presenting key pathways, cell types and signaling mechanisms involved in communication from the gut to the brain. We also illustrate mechanistic evidence relating to disorders of the gut which often have a central comorbidity component, such as in the case of inflammatory bowel diseases (IBD) and irritable bowel syndrome (IBS). Whilst research covering the central comorbidities associated with IBD and IBS continues to expand, the potential mechanisms linking neurological and gut changes following chemotherapy exposure remains under investigated.

### From the bottom-up

The GIT elicits a myriad of functions ultimately resulting in absorption of nutrients and expulsion of noxious chemicals and pathogens via muscular contractions, cellular, endocrine and immune mechanisms. Critically, the gut harbors a diverse microbial community (bacteria, fungi, archaea, viruses, and protozoa) and has prolific central effects mediating a healthy host (Feng et al., 2018). Consequently, changes in gut-microbial composition disrupts physiological homeostasis, often contributing to central maladaptations (Mu et al., 2016; Dinan and Cryan, 2017). Recent advances in our understanding of the impact the microbiota has on the gut-brain axis has led to common use of the term *microbiota-gut-brain axis* (Rhee et al., 2009; De Palma et al., 2014). Microbiota-gut-brain axis communication alters certain aspects of brain development, function, mood and cognitive processes from both the bottom-up and top-down (Catanzaro et al., 2014; De Palma et al., 2014; Mayer et al., 2014; Tillisch, 2014; Carabotti et al., 2015; Barbara et al., 2016). Evidence specifically related to chemotherapy-induced microbiota changes will be discussed further below (see reviews on microbiota-gut-brain axis; Rhee et al., 2009; De Palma et al., 2014; Mayer et al., 2014; Tillisch, 2014).

The GIT maintains an extensive intrinsic nervous system, the ENS which is unique in its ability to control certain functions of the small and large intestines even when it is disconnected from the CNS (Furness, 2016). However, the ENS should not be considered fully autonomous due to the constant top-down input it receives. The ENS is the largest and most complex division of the peripheral nervous system (PNS) comprising 400–600 million neurons and an extensive network of enteric glial cells (EGC) (Furness, 2012). EGCs share similarities with astrocytes, their CNS counterparts in the mechanisms they adopt to support enteric neurons, including their morphology, function and molecular capabilities (Gulbransen and Sharkey, 2009). Importantly, EGCs play key roles in mounting an immune response, particularly during intestinal inflammation.

Luminal environmental factors, such as mechanical and chemical changes are signaled from the gut to the brain via endocrine, immune and neuronal afferent pathways (Mayer, 2011; Furness, 2012; Al Omran and Aziz, 2014; Furness et al., 2014). Information regarding the level of distension, concentrations of specific nutrients, electrolytes, pH, and the presence of danger and immune signals is transmitted from the gut to the brain via a wide variety of neural and systemic communication pathways. Visceral changes are detected by a variety of sensory cell types including enterocytes, intrinsic and extrinsic primary afferent neurons, immune and enteroendocrine cells (Carabotti et al., 2015).

Hence, a wide variety of hormones and metabolites from the gut communicate homeostatic information to the brain via functional effector cells (enterocytes, smooth muscle cells, interstitial cells of Cajal, enterochromaffin cells, intrinsic and extrinsic primary afferent neurons, immune and enteroendocrine cells) (Carabotti et al., 2015). Examples of homeostatic information relayed from the functional effector cells include but are not exclusive to sensory, pH, water metabolism, chemical, danger and immune signals, etc.). Each cell type responds to luminal environmental changes and secretes specific signaling molecules which may include but are not exclusive to ghrelin, cholecystokinin, glucagon-like peptide-1, corticotrophin releasing hormone, proteases and cytokines, etc. (Furness et al., 2014). To further complicate gut-brain crosstalk, various neurotransmitters commonly produced centrally are also expressed in the GIT (Furness et al., 2014). Gut derived neurotransmitters, such as dopamine, serotonin and neuropeptide Y influence many aspects of central homeostasis, yet in the gut are responsible for appetite, satiety, hunger, pain and are implicated in the activation of reward pathways relating to food and beverage intake (Furness, 2016).

Numerous afferent and efferent pathways connect the gut and brain, presenting the host with a multitude of platforms for malfunction, dysregulation and disease, both in the periphery and centrally. Whilst the basic principles outlining top-down signaling have been extensively reviewed (Al Omran and Aziz, 2014; Furness et al., 2014; Furness, 2016) and is outside the scope of this review, it is crucial to acknowledge that these effects occur simultaneously with those described from the bottom-up. Importantly, top-down sympathetic and parasympathetic interactions suppress secretion, motility and GI transit, having direct effects on immune-, emotion-, mucosa-, and microflora-related alterations (Lyte et al., 2011; Mayer, 2011). Gut-brain axis dysfunction has played a pivotal role in our mechanistic understanding of various gut disorders and their effects on cognition. Indeed, experimentally induced gut disorders have critically developed our understanding of the mechanisms underlying central changes induced by disruptions in gut homeostasis. Disorders of the gut and chronic inflammation often result in psychological abnormalities, such as anxiety and depression (Nyuyki and Pittman, 2015). Additionally, physiological responses can be induced by stress, for instance triggering relapse in experimental colitis (Bernstein et al., 2010).

Great interest has recently been paid to the importance of gut health on mental health and vice versa. This has become particularly evident in the continual expansion of anecdotal evidence on the central comorbidities associated with various gut disorders, particularly in IBS and IBD (Drossman et al., 1999; Whitehead et al., 2002). Disorders of the gut are commonly associated with poorer mental health. For instance, 54–94% of IBS patients actively seeking treatment also present with emotional, psychological and cognitive comorbidities (Whitehead et al., 2002) as do chemotherapy recipients. The literature presented above provides clear evidence that gut disorders often occur simultaneously with central comorbidities, aligning with our hypothesis that gut-brain axis dysregulation may be mediating both chemotherapy-induced mucositis and neurological changes. Therefore, it is pivotal that we determine the *direct* and *indirect* central consequences of drug-induced gut disorders, such as chemotherapy-induced mucositis. Chemotherapy induces a range of peripheral and central side-effects, significantly reducing quality of life. In the gut this has been termed chemotherapy-induced mucositis and in the CNS, chemotherapy-induced cognitive impairment (CICI). The current review will now explore whether mucositis and CICI are linked and whether they exacerbate other symptoms, such as pain associated with mucositis, or cognitive impairment which are often experienced simultaneously in the chemotherapy setting.

## Chemotherapy from the bottom-up: the gut and cognition

**Figure 4:**
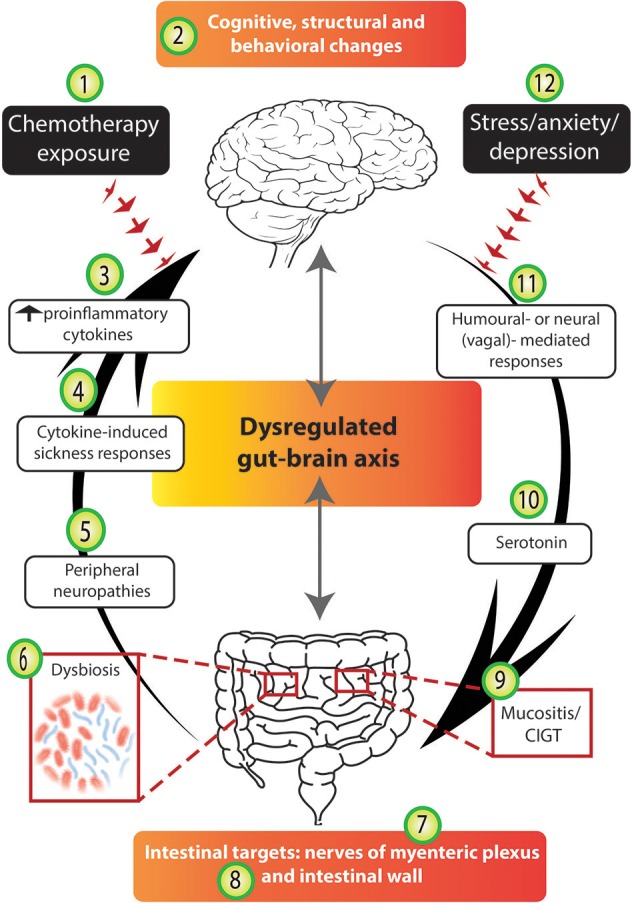
Chemotherapy disrupts several stages of the gut-brain axis. The arrows highlight the bidirectional nature of the gut-brain axis; in an unbalanced system mechanisms from the bottom-up (and vice versa) are disrupted. We suggest that chemotherapy-induced gut-brain axis dysregulation plays a major role in the intestinal, psychological and neurological complications experienced by many cancer patients. Chemotherapy exposure (1) often results in molecular and structural changes in the brain (2), e.g., hippocampal changes as identified in rodent models. Chemotherapy exposure causes cognitive and behavioral changes (2) to a subset of patients and these findings have been supported by some experimental models. The altered immune profile of chemotherapy recipients results in increased circulating pro-inflammatory cytokines (3) which have been reported to cause cytokine-induced sickness-like responses (4) which mimic chemotherapy-induced side-effects. Damage to peripheral nerves resulting in peripheral neuropathies (5) are experienced by some chemotherapy recipients. Chemotherapy targets the intestines and its microbial contents causing dysbiosis (6), impairing the nerves of the myenteric plexus (7), damaging intestinal wall parameters (8), and resulting in mucositis (9). Serotonin dysregulation under chemotherapy conditions (10) may play a role in chemotherapy-induced intestinal and neurological changes. Finally, both humoral and neural/vagal peripheral-to-central immune signaling pathways (11) may mediate chemotherapy-induced gut-brain axis dysregulation. Importantly, we acknowledge that the stress, anxiety and depression associated with cancer diagnosis and treatment (12) may contribute to both bottom-up and top-down pathways, having negative effects in the gut and the brain.

Chemotherapy drugs can be considered paradoxical at the most basic level. Primarily, they offer recipients' survivorship as they target malignant cells in an attempt to rid the host of cancer. On the other hand, due to their non-selective nature, they also target healthy cells and induce a range of side-effects reducing patient quality of life. The organ where their actions are perhaps often first noticed is the GIT due to its high regenerative capacity. Mucositis occurs in up to 70% of chemotherapy recipients and may manifest anywhere along the alimentary tract, termed oral or intestinal mucositis (Figure 4; Scully et al., 2003). It is one of the most significant dose-limiting side-effects of intensive anti-cancer therapy due to the painful nature of the disorder.

Sonis classified the pathogenesis of mucositis into five stages (Sonis, 2004). Hallmark characteristics of mucositis include villus atrophy, shallow crypts, inflammation and ulceration. Mucositis results in a high inflammatory response via the up-regulation and activation of various transcription factors, ultimately causing elevations in circulating proinflammatory cytokines (Figure 4), in particular IL-1β and TNF-α (Sonis, 2004). Whilst mucositis is an acute phenomenon which usually resolves upon cessation of chemotherapy treatment, clinical symptoms generally begin 5–10 days post-chemotherapy exposure and include significant pain, abdominal bloating, nausea and vomiting, diarrhea and/or constipation (Gibson and Keefe, 2006). Although guidelines for the prevention and treatment of mucositis exist, they fail to include effective treatment options (Gibson et al., 2013). Novel complementary treatment approaches are showing positive results utilizing naturally sourced products, such as Emu Oil and Rhubarb extract (Mashtoub et al., 2013; Bajic et al., 2016a). Although these treatment strategies show promise, to date they are still in the pre-clinical stages.

Our understanding of the central consequences of drug-induced gut disorders, such as mucositis remains elusive, yet evidence on CICI is expanding as various mechanisms underlying its pathogenesis are becoming clearer. CICI occurs in 15–45% of patients undergoing anti-cancer therapy (Figure 4; Vardy and Tannock, 2007). Subjective (self-report) report rates are considerably higher than objective measures with some studies reporting 95% of patients experiencing changes in cognitive performance (Downie et al., 2006). Subjective measures are nonetheless important as they identify the impact of cognitive impairment and the strain it places on patients' lives and daily functioning (Shilling and Jenkins, 2007). The breast cancer population forms the majority of the CICI literature as they offer researchers completion of extensive retrospective studies due to their typically good prognosis (Ahles et al., 2010). Regardless, CICI has been investigated in a range of other cancer types including myeloma and testicular cancer (Schagen et al., 2008; Potrata et al., 2010).

The cognitive domains most commonly reported in CICI are executive functioning, attention and concentration, processing speed, reaction time and motor speed and dexterity (Asher, 2011). Perceived cognitive impairment affects various facets of the patient's life, including relationships, employment, self-esteem/worth, finances and independence. The CICI experience leaves patients feeling distressed, anxious, frustrated, irritable, depressed and embarrassed, often reducing confidence (Mitchell, 2007; Von Ah et al., 2013). Current estimates on the duration of CICI are varied with some studies identifying deficits up to 20 years post-chemotherapy cessation, yet most indicate improvements up to 12 months later (Collins et al., 2009; Koppelmans et al., 2012). Neuroimaging studies have confirmed molecular and structural changes in the gray matter of the frontal and temporal lobes and the cerebellum of breast cancer patients following chemotherapy exposure (Silverman et al., 2007; McDonald et al., 2010). Additionally, chemotherapy induces white matter tract changes and the reorganization of global brain networks, which have undoubtable associative if not causal impacts on cognitive performance (Abraham et al., 2008; Bruno et al., 2012).

Animal studies have begun to unravel various mechanisms underlying the pathogenesis of CICI and involve structural and behavioral changes. Hippocampal and frontal cortical alterations have correlated with behavioral memory changes in various rodent models (Figure 4; Yang et al., 2010, 2012; Wigmore, 2012). Neurogenesis occurs in the dentate gyrus and cellular proliferation is critical in hippocampal circuit plasticity and memory consolidation (Deng et al., 2010; Ming and Song, 2011). CICI models have reported on the vulnerability of stem cells to proliferate in the dentate gyrus irrespective of chemotherapy drug class (Briones and Woods, 2011; El Beltagy et al., 2012). Considering the pivotal role neural stem cells in this region have to divide into new neurons or astrocytes, disruptions in this process present as a direct mechanism which may be contributing to CICI. More recently, neuroimmunological manifestations, such as glial dysregulation and neuroinflammation, have been reported to contribute to CICI (Briones and Woods, 2013; Bajic et al., 2016b).

Currently, effective prevention strategies or treatment approaches for CICI remain undetermined although two evidence-based guidelines are available to assist oncologists in addressing cognitive deficits (Network, 2015). Other interventions for CICI are broadly categorized into cognitive training, compensatory strategies, pharmacological, and complementary and integrative medicines (Vance et al., 2017). Recently, Toll-like receptors (TLRs) have been suggested as a common component in the pathology of neuropathy/pain and chemotherapy-induced gut toxicity, presenting a novel and much needed therapeutic approach in the treatment of chemotherapy-induced toxicities (Wardill et al., 2015). TLRs have profound homeostatic effects, tightly regulating innate immune and gut functions, modulating pain behaviors (Akira and Takeda, 2004; Rakoff-Nahoum et al., 2004; Doyle and O'Neill, 2006; Hutchinson et al., 2010, 2012; Gibson et al., 2016). Wardill et al. (2015) hypothesized that TLR-4 mediates glial activation and neuropathy driven by the molecular signals released from chemotherapy-induced gut toxicity. Primary studies have indicated that an altered TLR expression profile may contribute to chemotherapy-induced pain and diarrhea (Gibson et al., 2016). This study importantly highlights the need for further research examining both peripheral and central toxicities associated with chemotherapy treatment. Interestingly, the selective serotonin reuptake inhibitor, fluoxetine, has shown promising results in a rat model of CICI. Fluoxetine co-administration with the chemotherapy drug improved cognitive performance in rats assessed by object location recognition (Lyons et al., 2012). Whilst cellular proliferation in the dentate gyrus significantly reduced in the chemotherapy group, co-administration with fluoxetine reversed this reduction. Regardless of the evidence presented here indicating CNS changes following chemotherapy exposure, it is important to note that some studies have reported no structural or cognitive changes (Fremouw et al., 2012; Wilson and Weber, 2013). Various cytotoxic insults have revealed no morphological changes to neurons located in the CNS (Ginos et al., 1987; Gangloff et al., 2005). These negative findings could result from a range of factors, including differences in species, drug, dose, type of administration and cognitive parameters examined; but importantly, suggests that more complex mechanisms are likely to play a role in CICI.

Whilst the direct mechanisms presented here reflect the complex etiology of CICI, they fail to acknowledge the influence other simultaneously occurring side-effects may be having on CICI symptoms. Many of the CICI models described above utilized chemotherapy drugs that are also often used to examine mucositis, for example 5-Fluorouracil (5-FU), methotrexate and oxaliplatin. Although mucositis would have most likely been present in these models, the gut tissue would not have been examined and thus, the potential for *indirect* mechanisms relating to gut-brain axis dysregulation would have been ignored. In doing so, we may be missing critical mechanisms contributing to or exacerbating CICI pathogenesis. In order to explore this theory, we will now consider the influence chemotherapy exposure has on the gut-brain axis, opening novel hypotheses surrounding how mucositis may contribute to the etiology of CICI.

## Chemotherapy interrupts several stages of the gut-brain axis

As presented above, the two organs most vulnerable to the toxicities of chemotherapy treatment are the gut and the brain. Therefore, it is plausible that several stages of the gut-brain axis may become dysregulated in the chemotherapy setting (Figure 4). Here, we propose that chemotherapy exposure influences the gut-brain axis via several mechanisms which include: altering intestinal microbiota composition and function; upsetting the balance of “beneficial” and “detrimental” bacteria in the lumen, deleteriously affecting the gut lining, impairing the ENS and activating neuroimmune and pain signaling pathways (Figure 4). The interaction of the gut-brain axis and the neuropsychological comorbidities associated with specific gut disorders have been extensively reviewed, for example depression/cognitive deficits and IBS/IBD (Whitehead et al., 2002; Attree et al., 2003; Filipovic and Filipovic, 2014; Fond et al., 2014; Padhy et al., 2015). However, this angle of research is yet to be reviewed in the context of chemotherapy exposure and cognitive impairment. Research in this area will continue to develop as we begin to appreciate that chemotherapy-induced side-effects involving the gut-brain axis may continue to linger for some time after treatment cessation, placing significant strain on health care and importantly, patient quality of life.

### The microbiome

It has been estimated that our gut contains 100-fold more genes than the human genome and approximately 1,000 bacterial species (Ley et al., 2006; Qin et al., 2010). Our gut microbiome coevolves with us (Ley et al., 2008) and changes may be either beneficial or detrimental to human health. In healthy individuals, the gut microbiota is responsible for a number of health benefits, such as pathogen protection, nutrition, host metabolism and immune modulation (O'Hara and Shanahan, 2006). Although a core microbial population has been established in individuals, changes can be caused by many factors including age, diet, antibiotic and analgesic use and environmental factors (Jalanka-Tuovinen et al., 2011). The microbiome facilitates intestinal homeostasis and more specifically, has the capacity to influence inflammation and immunity, both at the local (mucosal) and systemic levels (Clemente et al., 2012). Commensal bacteria play important roles in anti-viral immunity, regulating systemic immune activation (Abt et al., 2012). Signals released by commensal bacteria assist in immune development and thereby, have important implications for infectious and inflammatory disease susceptibility (Ichinohe et al., 2011; Abt et al., 2012), such as in the case of chemotherapy-induced mucositis. Consequently, dysbiosis can heavily influence pathological intestinal conditions with an inflammatory component, for example in experimentally-induced IBD (García-Lafuente et al., 1997; Dalal and Chang, 2014; Touchefeu et al., 2014; Håkansson et al., 2015). Critically, IBD patients reported microbial composition changes with major shifts in genomic landscape and functional outcomes (Morgan et al., 2012). Undoubtedly, the implications of such IBD studies have heavily impacted oncology, raising many questions specifically relating to the intestinal microbiota, immune, malignancy and anti-cancer treatment interactions.

Whilst Sonis' five-phase model of mucositis (Sonis, 2004) lacked any potential influence on the microbiota, unequivocal research has indeed confirmed that intestinal inflammation modulates microbiome composition and function (Morgan et al., 2012; Touchefeu et al., 2014). As intestinal inflammation is a common characteristic of mucositis, it makes sense that chemotherapy induces functional and compositional changes to the microbiome. It has been suggested that mucositis development is influenced by commensal bacteria in multiple pathways involving inflammation and oxidative stress, intestinal permeability (discussed below), mucus layer composition, epithelial repair mechanisms and via the release of immune effector molecules (van Vliet et al., 2010). Indeed, research has begun to unravel the complexities surrounding the interactions between the host and the intestinal microbiota following chemotherapy exposure and consequently, several excellent reviews exist (van Vliet et al., 2010; Touchefeu et al., 2014; Dzutsev et al., 2015; Vanhoecke et al., 2015; Zitvogel et al., 2015). Commonly used chemotherapy drugs, such as 5-FU and irinotecan report drastic shifts in intestinal microflora, from commensal bacteria which maintain a symbiotic relationship with the host, to elevated levels of *Escherichia* spp., *Clostridium* spp., and *Enterococcus* spp. which can be associated with several pathologies involving inflammation and infection (Von Bültzingslöwen et al., 2003; Stringer et al., 2007, 2009; Lin et al., 2012; Table 1). Several clinical studies have supported pre-clinical findings describing alterations in fecal microbial composition following chemotherapy treatment. Literature reveals a general decrease in the overall diversity of bacteria in the microbiota of cancer patients undergoing anti-cancer treatment when compared to healthy individuals, irrespective of cancer type or chemotherapy regime (Manichanh et al., 2008; Zwielehner et al., 2011; Montassier et al., 2014; see Table 1).

**Table 1:** Summary of key papers highlighting chemotherapy-microbiota-immune interactions.

**Study**	**Subjects**	**Treatment**	**Commentary**
Lin et al., 2012	Tumor bearing rats	Irinotecan alone Irinotecan/5-FU	Increased abundance *clostridial clusters I, XI*, and *Enterobacteriaceae*.
Von Bültzingslöwen et al., 2003	Rats	5-FU	Increased facultative and anaerobic bacteria from the oral cavity. Increased facultative anaerobes in large intestine. Proportion of facultative gram-negative rods increased in both oral cavity and intestine.
Stringer et al., 2009	Rats	Irinotecan	Increased jejunal samples of *Escherichia* spp., *Clostridium* spp., *Staphylococcus* spp. Increased colonic samples of *Escherichia spp*., *Clostridium spp*., *Enterococcus spp*., *Serratia spp*., *Staphylococcus spp*. No changes in fecal flora except *E. coli*.
Stringer et al., 2007	Rats	Irinotecan	Extensive changes were evident in stomach, jejunum, colon and feces. Most significant changes were in colon, indicating a relationship between colon bacteria modification and diarrhea incidence.
Montassier et al., 2014	Patients with non-Hodgkin's lymphoma	Bone marrow transplantation with chemotherapy conditioning	Steep reduction in alpha diversity during chemotherapy. Decreases in *Firmicutes bacteria* and *Bifidobacterium* whilst *Bacteriodes* and *Esterichia* were increased
Manichanh et al., 2008	Patients with abdominal tumors	Pelvic radiotherapy	Faecal samples reported significant microbiota profile changes in patients with post-radiotherapy diarrhea. Not all patients reported diarrhea. Importantly, this study suggests initial microbial colonization may be linked to susceptibility or protection against diarrhea following radiotherapy treatment.
Zwielehner et al., 2011	Patients with various malignancies	Chemotherapy and antibiotic treatment	Chemotherapy decreased *Clostridium cluster IV* and *XIVa*. *C. difficile* was present in three out of seventeen patients and was accompanied by a decrease in the genera *Bifidobacterium, Lactobacillus, Veillonella* and *Faecalibacterium prausnitzii*. *Enterococcus faecium* increased following chemotherapy.
Iida et al., 2013	Tumor bearing mice	Oxaliplatin and cisplatin	Chemotherapy-induced dysbiosis impairs response to immunotherapy and chemotherapy.
Viaud et al., 2013	Tumor bearing mice	Cyclophosphamide	Jejunal and fecal samples reported dysbiosis and induces translocation of specific Gram-positive bacteria to secondary lymphoid organs whereby they stimulate subsets of T cells. These results suggest that the gut microbiota may affect anticancer immune response.
Sivan et al., 2015	Tumor bearing mice	Co-housing, fecal transfer, programmed cell death protein 1 ligand 1 (PD-L1)–specific antibody therapy (checkpoint blockade), oral *Bifidobacterium*	Changes to anti-tumor immunity were eliminated by co-housing and fecal transfer. Oral *Bifidobacterium* administration improved tumor control to same degree as PDL-1 therapy; combination treatment nearly abolished tumor outgrowth.
Vétizou et al., 2015	Tumor bearing mice and metatstatic melanoma patients	Ipilimumab (CTLA-4 blocker) regulates T cell activation and improves survivability of metastatic melanoma patients.	CTLA-4 blockade is influenced by the microbiota. Changes in *B. fragilis* and/or *B. thetaiotaomicron* and *Burkholderiales* affects immune response facilitating tumor control in mice and patients.

In addition to the direct effects microorganisms and their enzymes have on cancer initiation and progression (Sears and Garrett, 2014; Gagnière et al., 2016), the microbiota also modifies drug absorption and metabolism via gene expression changes (Carmody and Turnbaugh, 2014; Wilson and Nicholson, 2017). This has become a pivotal research angle in oncology as chemotherapy-microbiota-immune interactions have identified microbial-mediated innate and adaptive immune responses and their effect on the efficacy of cancer immunotherapy and chemotherapy drugs (Iida et al., 2013; Viaud et al., 2013; Sivan et al., 2015; Vétizou et al., 2015). Two crucial studies in *Science* Iida et al. (2013); Viaud et al. (2013) reported that microbiota disruption by antibiotic treatment impaired chemotherapy drug efficacy on tumors, utilizing cyclophosphamide and oxaliplatin. More recent studies have illustrated the important role certain microbial strains (*Bifidobacterium*) play in anti-tumor immunity (Sivan et al., 2015; Vétizou et al., 2015). Although these studies were performed in mice, their findings indicate the potential risks associated with the use of antibiotics during chemotherapy treatment. The growing field of microbiome research has raised a lot of questions and comments on the complex interplay and interwoven relationships between microbes and cancer, including anti-cancer treatments (Pennisi, 2013; Bordon, 2014; Greenhill, 2014; Lokody, 2014; Mukaida, 2014). Further, some of the above studies (Sivan et al., 2015; Vétizou et al., 2015) have implications for microbial therapy in cancer immunotherapy. As our understanding of these interactions continues to progress, new knowledge in this area will open up possibilities of novel paradigm shifts in treatment approaches which may improve anticancer efficacy and even prevent toxicity. The studies presented in this section suggest a role for chemotherapy-induced dysbiosis in intestinal disease pathogenesis and chemotherapy-induced gut-brain axis dysregulation (Figure 4). As mentioned, commensal bacteria are critical in regulating intestinal homeostasis and more specifically, intestinal integrity. In fact, the effects commensal bacteria have on intestinal integrity and vice versa, go hand-in-hand. Accordingly, the reciprocal relationship shared between commensal bacteria and the intestinal wall will be presented together in the following section. Chemotherapy compromises intestinal integrity and leads to profound effects on the gut lining, eventually leading to a dysbiotic microbial community and consequently risking microbial invasion into the systemic circulation.

### Chemotherapy impairs intestinal barrier-microbiota interactions

The even comprehensively described pathogenesis of mucositis (Sonis, 2004) is unable to fully encapsulate the mechanisms underlying the pathogenesis of chemotherapy-induced gut damage. Although it covers many essential aspects of the pathological processes underlying mucositis, such as epithelial barrier damage. More recently, some research groups have re-defined gut damage caused by chemotherapy as chemotherapy-induced gut toxicity (CIGT). The proposed term includes additional pathological manifestations caused by chemotherapy treatment, such as abnormalities in tight junctions, immune dysfunction and microbiota influences (Montassier et al., 2014; Touchefeu et al., 2014; Wardill et al., 2014).

Nonetheless, the epithelial barrier lining of the GIT is fundamental in ensuring the maintenance of intestinal integrity. As well as forming a mechanical barrier to separate the inside of the body from the outside world, it is heavily involved in the communication shared between the body and the intestinal contents (Powell, 1981). Tight junctions are intertwined throughout the epithelial barrier and regulate diffusion of solutes according to strict size and charge limitations (Balda and Matter, 2016). Chemotherapy exposure increases intestinal permeability and the most widely studied mechanisms to date have included apoptosis of intestinal crypts and villous atrophy (Keefe et al., 2000; Carneiro-Filho et al., 2004). Early clinical studies assessing the severity of intestinal damage in high dose regimes reported abnormalities in intestinal permeability and defects in tight-junction integrity (Figure 4; Keefe et al., 1997). Convincing rodent evidence has implicated mucosal barrier injury and tight junction deficits with gut toxicity induced by various chemotherapy drugs, including irinotecan and methotrexate (Beutheu Youmba et al., 2012; Wardill et al., 2014). However, it should be noted that rodent model application in gut immunity and microbiome research has serious limitations and pitfalls due to compositional microbiota differences between species. Whilst the rodent microbiome shares some common features with human commensal bacteria, unique commensals in rodents have differential effects in immune responses and disease pathogenesis (Nguyen et al., 2015). Consequently, animal models of inflammation are different to human models of inflammation in terms of microbial colonization, morphology of lesions and clinical manifestations. Nonetheless, research in experimental models continues to provide critical insight into complex interactions between the host, microbiota and immune responses. More recently, it has been becoming more evident the impact intestinal integrity has on the microbiota and vice versa, especially under chemotherapy conditions. The health and stability of the intestinal wall influences the microbiota and vice versa.

Commensal bacteria in the microbiota have a protective effect on intestinal integrity, interacting with TLR and Nuclear Factor kappa B pathways, ensuring the development of an innate immune response (Doyle and O'Neill, 2006). These components of innate immunity in the gut and the activation of these pathways are pivotal in maintaining barrier function, promoting mucosal repair and protecting the gut against injury (Rakoff-Nahoum et al., 2004; Cario, 2008). Chemotherapy exposure alters commensal microbial composition in the microbiota, thus negatively affecting barrier function, repair pathways and compromising intestinal integrity (Stringer et al., 2009). Accordingly, further investigations are required to fully appreciate the role chemotherapy-induced intestinal permeability changes play in gut-brain axis dysregulation. As intestinal integrity becomes compromised under chemotherapy conditions, it is not surprising that nerves of the myenteric plexus and peripheral nerve endings become damaged as these neural components also reside outside of the blood-brain barrier (BBB) as will be discussed below.

### Chemotherapy results in peripheral and enteric neuropathy

The PNS is particularly vulnerable to the cytotoxic nature of different chemotherapy drug classes, including platinum analogs, antitubulins, proteasome inhibitors, immunomodulatory agents and some newer biologics, such as brentuximab (Cavaletti and Marmiroli, 2015). Chemotherapy-induced peripheral neuropathy (CIPN) is experienced by 30–40% of chemotherapy recipients and is often responsible for early cessation of treatment, decreasing chemotherapeutic efficacy and causing higher relapse (Wang et al., 2012; Areti et al., 2014). Typically, sensorimotor symptoms are more common than motor involvement, presenting in a bilateral “glove-and-stocking” distribution in the hands and feet to include paraesthesia, numbness, burning pain, allodynia and hyperalgesia (Windebank and Grisold, 2008). However, the development of motor and autonomic neuropathic symptoms may also occur, such as sensory ataxia, pain, weakness of distal muscles, reduced deep tendon reflexes and severe numbness that can severely affect the patient's ability to function and their quality of life (Park et al., 2013). Often symptoms fail to improve after cessation of treatment, referred to as a “coasting” phenomenon (Windebank and Grisold, 2008).

The pathogenesis of CIPN is primarily related to axonopathy and neuronopathy in which dorsal root ganglia (DRG) are involved. Peripheral nerves and their ganglia are particularly susceptible to chemotherapy-induced damage due to their location as they lack the protective defenses associated with the BBB (Furness et al., 2014). For example, chemotherapy interrupts the cell cycle, inducing structural and functional changes in DRG which partly explain the development of sensory symptoms in CIPN (Gill and Windebank, 1998; Cavaletti et al., 2000). Many pathophysiological mechanisms mediating chemotherapy-induced peripheral nerve damage have been identified. Some examples include, but are not exclusive to dysregulated axonal transport and trophic factor support via microtubule structural changes (Theiss and Meller, 2000), mitochondrial stress (McDonald and Windebank, 2002; Chen et al., 2007) and reduced blood supply to nerves (Theiss and Meller, 2000; Isoardo et al., 2004). Further changes contributing to CIPN pathogenesis include dysregulated ion channels, neurotransmitter release and receptor sensitivity (Descoeur et al., 2011; Mihara et al., 2011; Tatsushima et al., 2011). The evidence presented here clearly describes mechanisms by which the PNS is damaged following chemotherapy exposure, forming an important element of the proposed central hypothesis (Figure 4).

In addition to peripheral neuropathies, neurons residing within the ENS are also susceptible to the deleterious effects of various chemotherapy drugs, including cisplatin, oxaliplatin and more recently, 5-FU (Vera et al., 2011; Wafai et al., 2013; McQuade et al., 2016). Systemic administration of these chemotherapy drugs induces structural and functional changes to myenteric neurons (Figure 4), consequently resulting in downstream negative effects on GI motility. Interestingly, acute exposure of 5-FU increases intestinal transit whilst prolonged treatment decreases transit time (McQuade et al., 2016). These findings outline the complex nature chemotherapy drugs have on enteric neurons and altered motility patterns. Here, we highlight that chemotherapy results in damage to neurons and ganglia residing outside of the BBB, exerting functional maladaptations in both the PNS and ENS. So far we have described several mechanisms relating to chemotherapy-induced gut-brain axis dysregulation, yet we have not identified how immune signals from the intestinal cavity may communicate to the brain and potentially contribute to the pathogenesis of CICI. In the following section we present peripheral-to-central immune pathways as being critical in the transmission of signals from the gut to the brain following chemotherapy exposure.

### Peripheral-to-central immune signaling pathways mediating chemotherapy-induced gut-brain axis dysregulation

Historically there has been controversy surrounding the theory that a communication system existed between the immune system and the CNS (Dantzer and Kelley, 2007). Traditionally it was assumed that proinflammatory cytokines were unable to pass through the BBB due to their size. However, the humoral route explained that cytokines expressed in the periphery could in fact cross the BBB at leaky circumventricular organs through fenestrated capillaries. At these sites blood-borne cytokines act on parenchymal astrocytes that express secondary mediators, such as nitric oxide and prostaglandins which freely diffuse to nearby brain regions, such as the hypothalamus to mediate the effects of pyrogenic and corticotropic cytokines (Katsuura et al., 1990). Whilst this hypothesis leads toward the existence of a communication system between the immune system and the CNS, it was unable to fully account for other contributing pathways that may be mediating physiological responses. Consequently, it is now widely accepted that peripheral cytokines signal the brain and in turn, this triggers sickness behavior responses (Dantzer, 2004).

Central or peripheral immune challenges trigger a range of physiological, behavioral and motivational changes to assist the host in healing (Figures 2, 4). Non-specific symptoms which accompany sickness behaviors include, but are not exclusive to fever, depressed activity, a loss of interest in regular activities (appetite, sexual, cleaning, hygiene), weakness, malaise, listlessness and cognitive changes (Dantzer and Kelley, 2007). As demonstrated by Dantzer and Kelley (Dantzer and Kelley, 2007), the last two decades of research on this phenomenon have confirmed that local or systemic proinflammatory cytokines expressed at physiological levels, during both acute and chronic inflammatory responses, serve as true communication molecules between the immune system and brain. For example, direct administration of IL-1β or TNF-α to the lateral ventricle decreased social exploration and feeding behavior in rats (Kent et al., 1992). In the chemotherapy setting, this phenomenon may be related to either the systemic nature of the drugs themselves or localized inflammatory responses occurring as a result of the toxicities associated with their use, such as in the case of mucositis (see Figure 2).

Interestingly, whilst IL-1β and TNF-α are key proinflammatory cytokines instigating sickness behavior responses, they also play a pivotal role in the pathogenesis of mucositis. Since both proinflammatory cytokines play an important role in the pathogenesis of mucositis and sickness behaviors which involve cognitive deficits, it is plausible that these cytokines and the pathways mediating their activation may present as key mechanisms underlying the central hypothesis in this review (Figure 4). Various animal models have identified that sickness behavior responses may be induced by a range of clinical conditions, such as systemic or central administration of lipopolysaccharide (active fragment of gram negative bacteria) or recombinant proinflammatory cytokines (Tomas et al., 1984; Goehler et al., 1999; Dantzer and Kelley, 2007). Furthermore, many symptoms associated with cytokine-induced sickness responses mimic the cluster of chemotherapy-induced side-effects, including fatigue, depression, reduced appetite, heightened sensitivity to pain and cognitive impairment (Figure 4). As previously mentioned, up to 70% of chemotherapy recipients experience mucositis (Scully et al., 2003) and an altered immune profile due to the systemic nature of anti-cancer treatments, yet whether these side-effects may be contributing to CICI remains elusive. Accordingly, we present pathways which may be enabling the communication of peripheral immune signals to the brain, more specifically defining how mucositis-driven inflammation may signal the brain via vagal- and neural-mediated mechanisms and contribute to the pathogenesis of CICI.

Information from proinflammatory cytokines and mediators expressed under chemotherapy-induced mucositis conditions may signal the brain via a vagal communication pathway (Figure 4). Dendritic cells (DCs) are a specialized subset of immune cells located within the vagus nerve and surrounding paraganglia (Goehler et al., 1999). The signals (proinflammatory cytokines, chemokines and mediators) expressed by DCs are capable of communicating to the brain (Banchereau and Steinman, 1998; Reis e Sousa et al., 1999). Vagal immunosensation requires primary afferent neuron activation as the initial interface triggering the brain. Following chemotherapy exposure, proinflammatory cytokines and mediators, such as those from the IL-1 family arise from mucositis-induced inflammation. IL-1 binds to receptors on the paraganglia surrounding vagal afferents and release neurotransmitters onto the vagus, consequently activating vagal fibers. A vagal-mediated neural signal is then carried to the nucleus tractus solitarius which projects the message to higher order brain regions, such as the hypothalamus and hippocampus, whereby, IL-1 production is increased and other neural cascading events are initiated to produce sickness behavior responses (Dantzer and Kelley, 2007; Wardill et al., 2015). Whilst this evidence clearly demonstrates the role that vagal afferent nerves play in peripheral-to-central transmission of immune messages from the abdominal cavity, to date these pathways have not been examined under chemotherapy conditions and are therefore presented as potential mechanisms contributing to gut-brain axis dysregulation.

DCs play a role in immunomodulation and neuroimmune regulation, crucially bridging innate and immune adaptive processes. Importantly, DCs express pattern recognition receptors for a range of chemicals (e.g., TLRs), chemokines, microorganisms and neurotransmitters, such as serotonin (Banchereau and Steinman, 1998; Reis e Sousa et al., 1999). Damage to surroundings GIT tissues and increased levels of proinflammatory mediators, such as cytokines and chemokines induce maturation of DCs (Ricart et al., 2011). Matured DCs migrate to secondary lymphoid organs to initiate a localized immune response via interacting with naïve T cells (Banchereau and Steinman, 1998). Recent data implies an emerging role for DC-expressed serotonin and receptor activation in regulating innate and immune responses associated with gut inflammatory conditions (Holst et al., 2015; Szabo et al., 2018). Mechanisms underlying DC-mediated serotonin and receptor-sub types affect various levels of localized inflammation, even having anti-inflammatory effects preventing excess inflammation and tissue damage (Szabo et al., 2018). These findings coupled with the aforementioned positive cognitive effects of fluoxetine in a rat model of CICI (Lyons et al., 2012), serotonin presents as a new therapeutic approach for inflammatory disorders, having effects in both the gut and the brain. Accordingly, serotonin is presented as an underestimated contributing factor potentially implicated in chemotherapy mediated gut-brain axis dysregulation (see Wigmore, 2012).

## Conclusion

From the bottom-up, the gut and the brain are the two primary organs most susceptible to toxicity associated with the non-selective nature of chemotherapy drugs. As chemotherapy exposure induces cognitive decline and mucositis in a subset of recipients, it makes sense that several stages of the gut-brain axis are prone to negative effects in this setting. The gut-brain axis is largely responsible for the maintenance of homeostasis and achieves this delicate balance by integrating a vast array of signals and information from many systems, as described above and shown in the figures. In this regard, upsetting the balance of any stage in the gut-brain axis following chemotherapy treatment has the potential to exacerbate side-effects, such as in the case of mucositis and CICI. The findings from our review support our main hypotheses that chemotherapy treatment causes severe and prolonged psychosocial impacts on the survivor. Secondly, the gut-brain axis is an important mediator of a diverse range of cognitive and emotional disorders similar to those experienced by cancer survivors. Evidently, chemotherapy affects the gut-brain axis at several key stages which are outlined above. Collectively, we conclude that the psycho-social side-effects of chemotherapy treatment may be caused by the effects of chemotherapy on the gut-brain axis.

Apart from chemotherapy treatments crossing the BBB and *directly* causing damage to specific regions, peripheral inflammatory responses from either the malignancy or systemic treatment also *indirectly* cause cellular changes in the spinal cord. We recently demonstrated glial dysregulation in the thoracic region of rats with 5-FU-induced intestinal mucositis indicating an indirect regional-specific neuroimmune response to CIGT (Bajic et al., 2015). Our data provides evidence that experimentally-induced jejunal toxicity *indirectly* downregulates thoracic astrocytic expression. In addition to this recent finding, the evidence presented here suggests a role for chemotherapy-induced dysbiosis in intestinal inflammation. This further complicates intestinal inflammation and ulceration induced by chemotherapy exposure which may potentially influence CICI. Neurons in both the ENS and the PNS are also vulnerable to the cytotoxic nature of chemotherapy treatments. The implications of co-administration of pharmacological interventions (e.g., fluoxetine) with chemotherapy drugs remains undetermined, although preliminary studies showing improvements in cognitive performance warrants further investigation. In view of the aforementioned data, we conclude that several stages of the gut-brain axis become dysregulated following chemotherapy exposure and may be implicated in the pathogenesis of CICI. Harnessing our understanding of the role gut-brain axis dysregulation plays in modulating brain function may offer clues for more targeted therapeutic strategies to prevent CICI and warrants further investigation.

## Author contributions

JB wrote this manuscript to form part of her thesis and it is her second review. IJ, GH, and MH contributed equally at various points throughout the course of this manuscript, adding valuable input and critically reviewing each draft made by JB.

### Conflict of interest statement

The authors declare that the research was conducted in the absence of any commercial or financial relationships that could be construed as a potential conflict of interest.

## Abbreviations

CICI: Chemotherapy-induced cognitive impairment
ENS: enteric nervous system
PNS: peripheral nervous system
EGC: enteric glial cells
IBS: irritable bowel syndrome
IBD: inflammatory bowel diseases
FGIDs: functional gastrointestinal disorders
BBB: blood-brain barrier
IL-1β: interleukin-1β
IL-6: interleukin-6
TNF-α: tumour necrosis factor alpha
5-FU: 5-Fluorouracil
DSS: dextran sodium sulfphate
CIGT: chemotherapy-induced gut toxicity
TLRs: toll-like receptors
CIPN: chemotherapy-induced peripheral neuropathy
5-HT: serotonin 5-hydroxytryptamine
DCs: dendritic cells.
